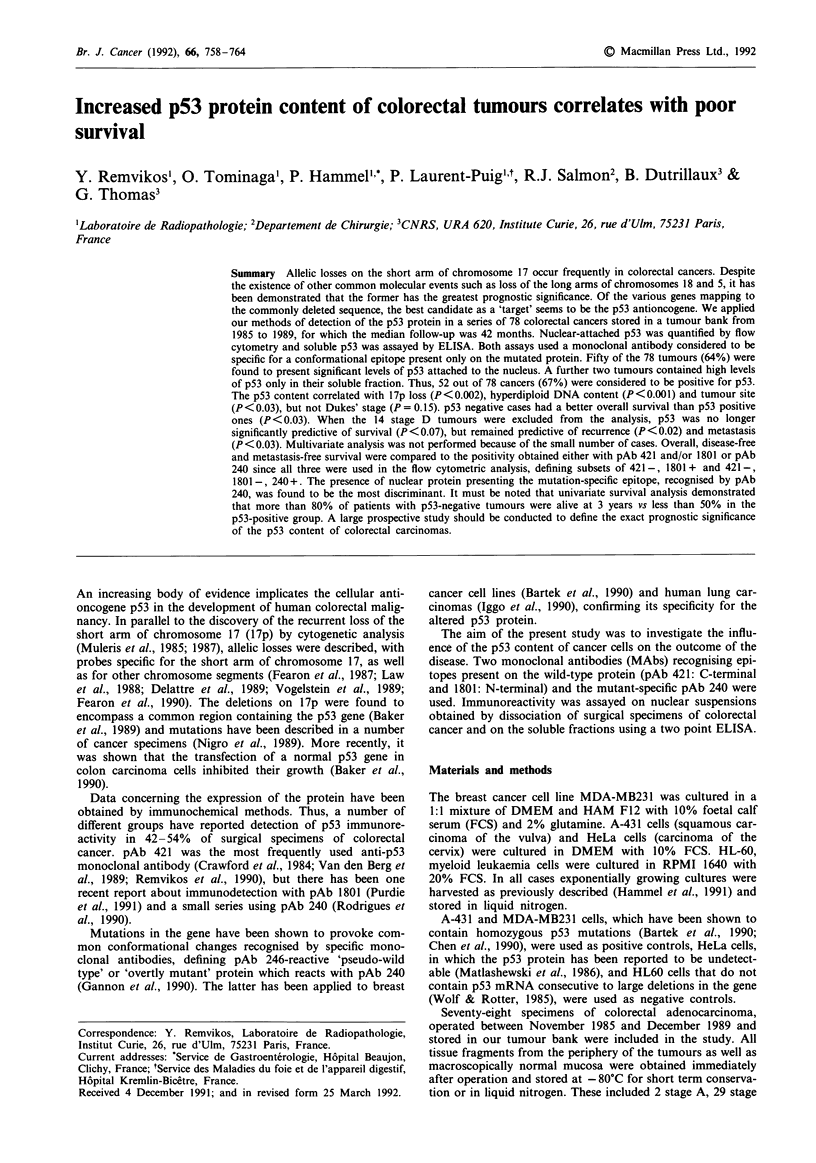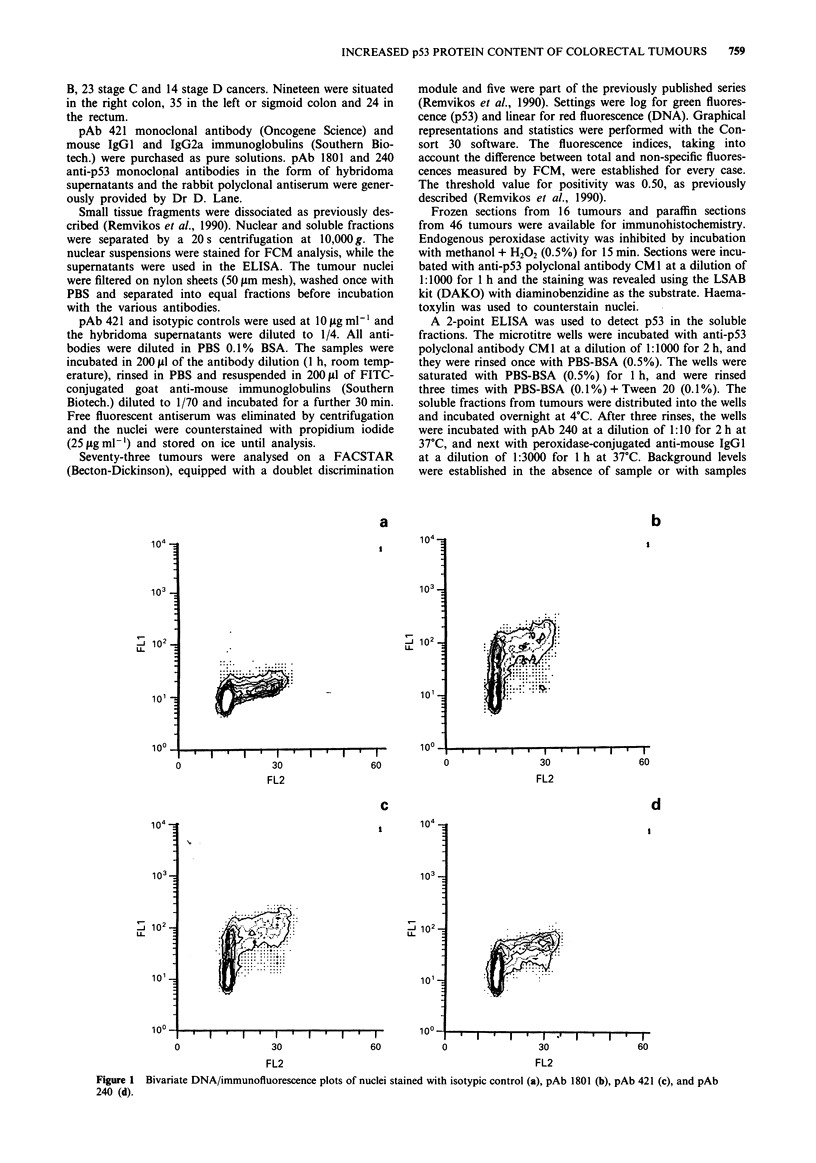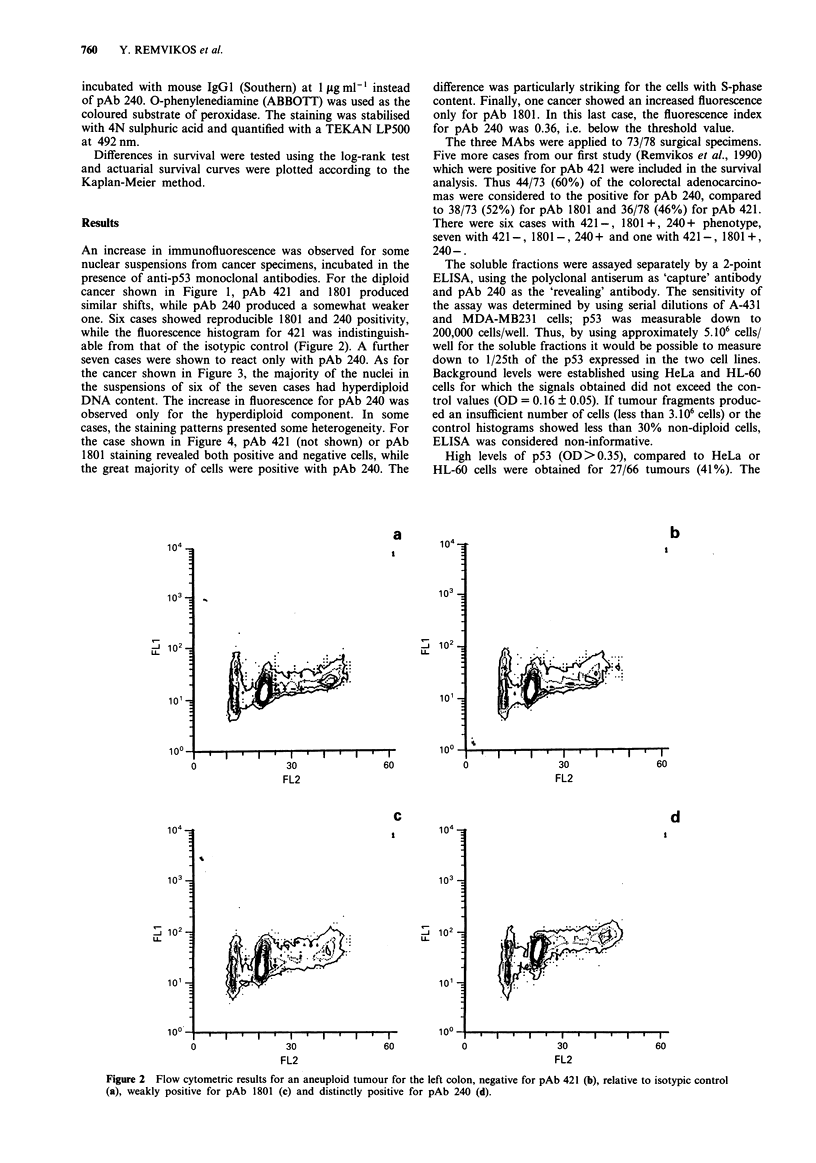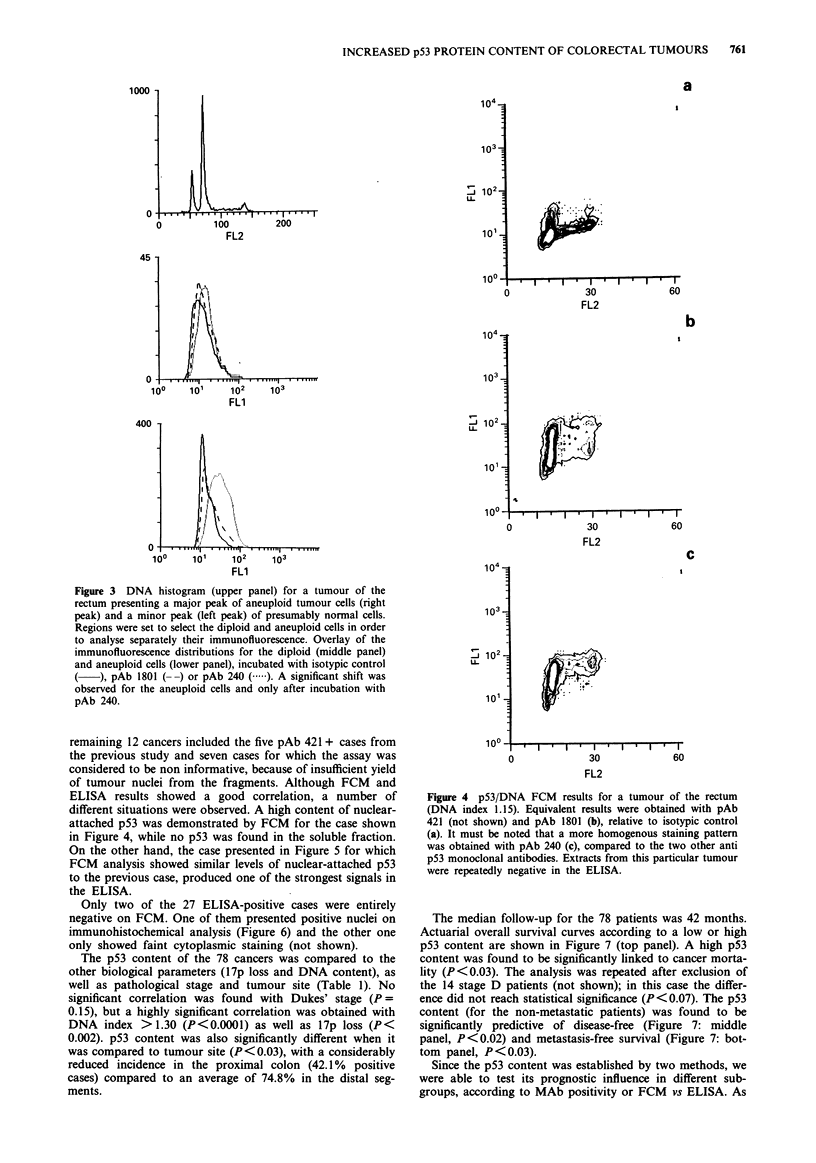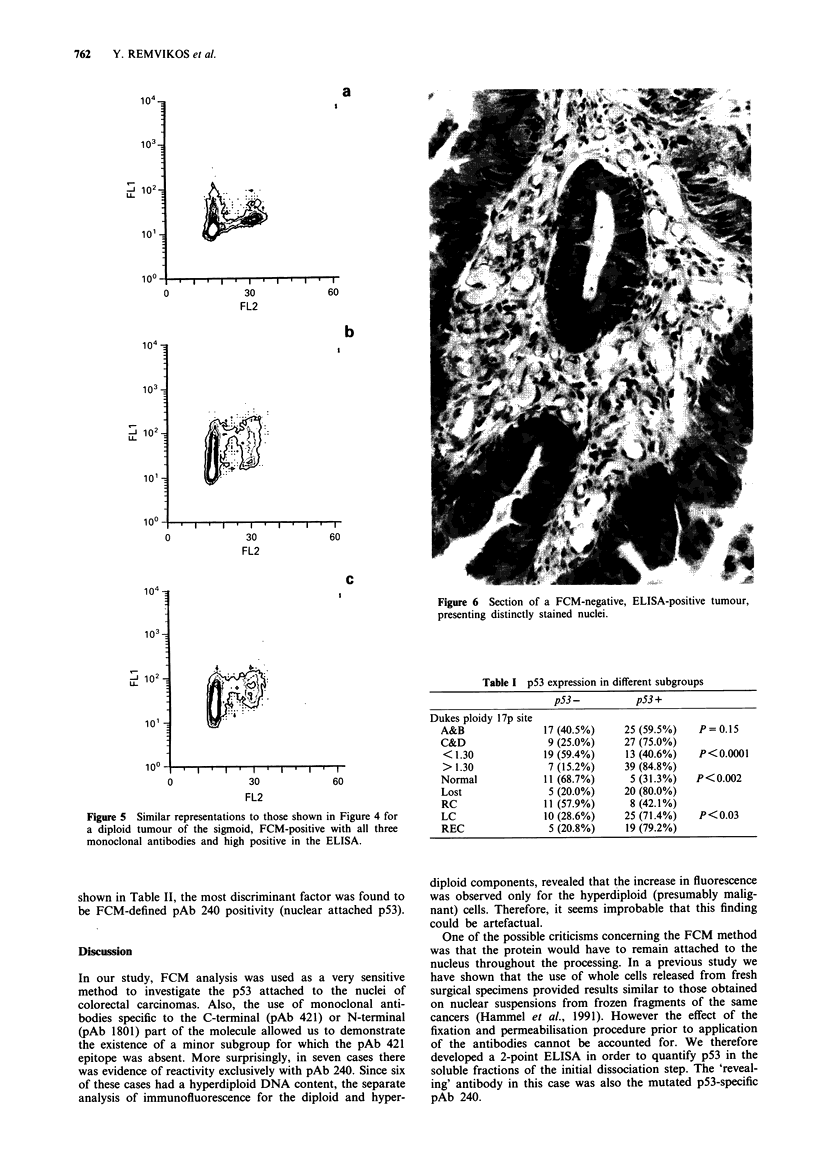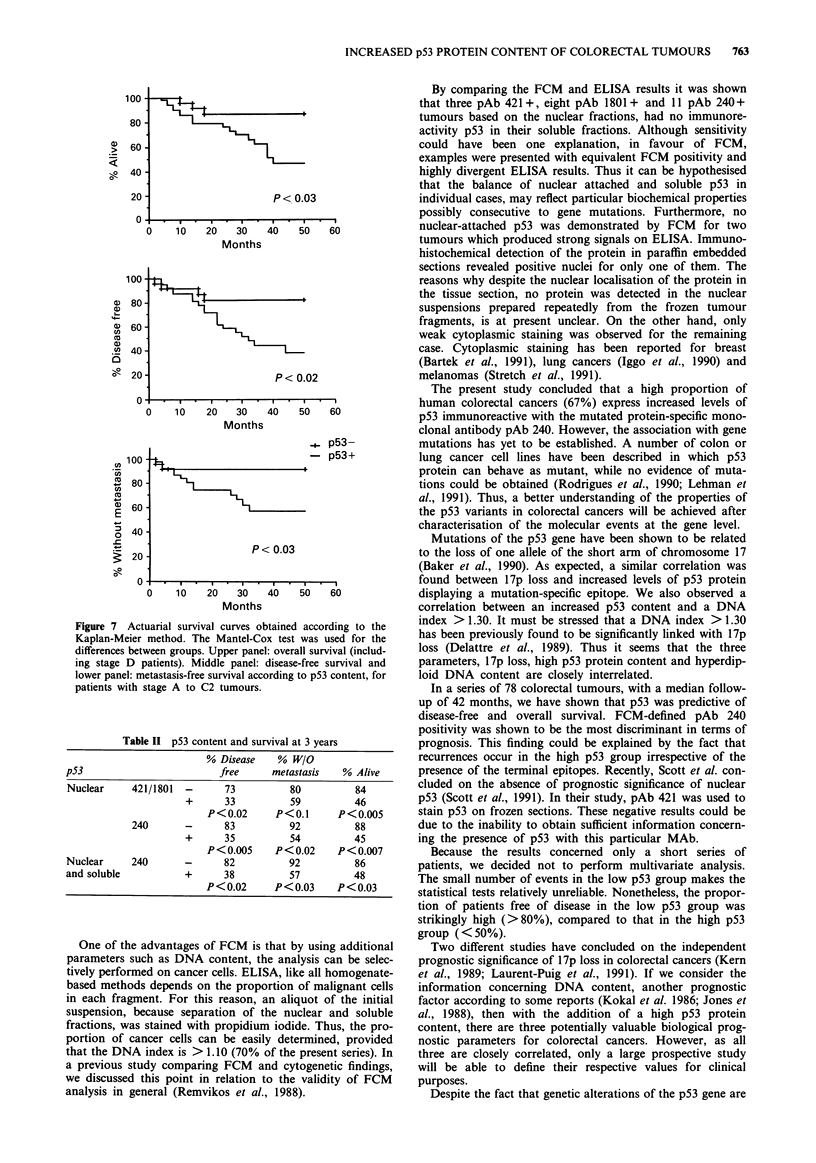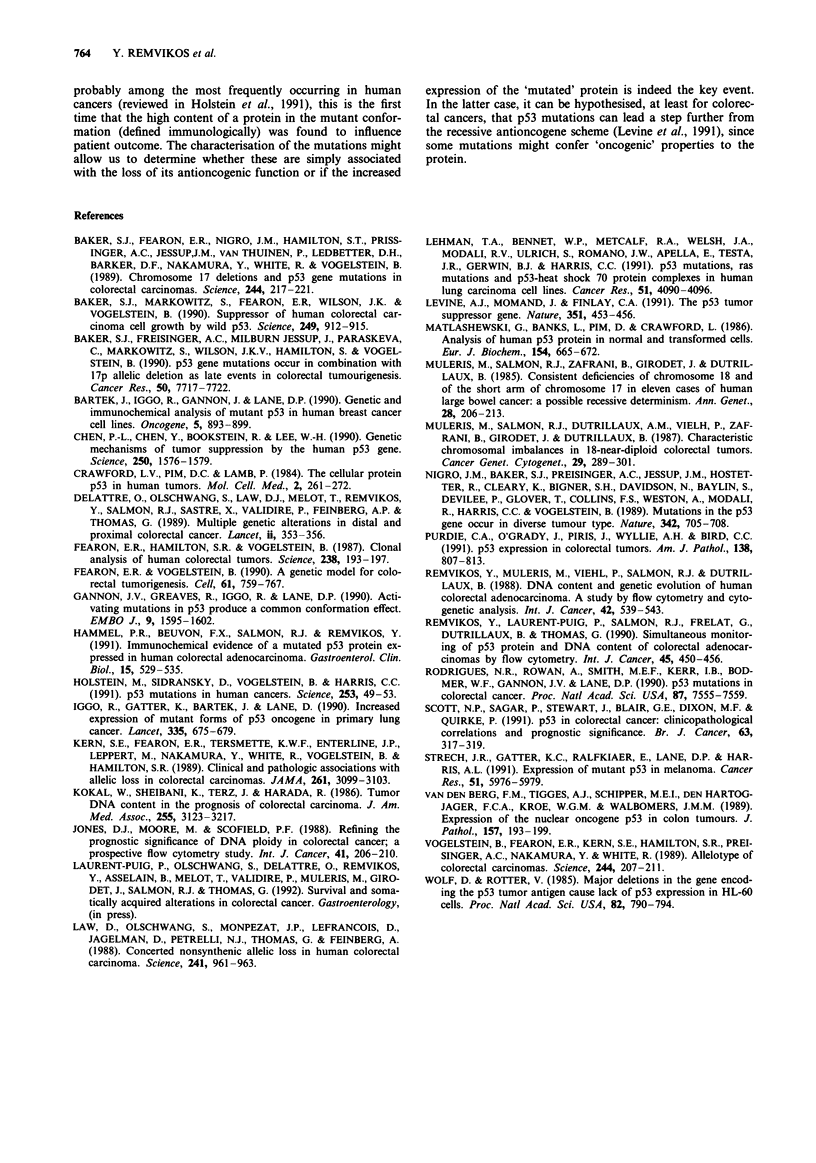# Increased p53 protein content of colorectal tumours correlates with poor survival.

**DOI:** 10.1038/bjc.1992.352

**Published:** 1992-10

**Authors:** Y. Remvikos, O. Tominaga, P. Hammel, P. Laurent-Puig, R. J. Salmon, B. Dutrillaux, G. Thomas

**Affiliations:** Laboratoire de Radiopathologie, URA 620, Institute Curie, Paris, France.

## Abstract

**Images:**


Br. J. Cancer (1992), 66, 758–764 © Macmillan Press Ltd., 1992

### Increased p53 protein content of colorectal tumours correlates with poor survival

Y. Remvikos[1], O. Tominaga[1], P. Hammel[1,*], P. Laurent-Puig[1,†], R.J. Salmon[2], B. Dutrillaux[3] & G. Thomas[3]

[1]*Laboratoire de Radiopathologie;* [2]*Departement de Chirurgie;* [3]*CNRS, URA 620, Institute Curie, 26, rue d'Ulm, 75231 Paris, France*

**Summary** Allelic losses on the short arm of chromosome 17 occur frequently in colorectal cancers. Despite the existence of other common molecular events such as loss of the long arms of chromosomes 18 and 5, it has been demonstrated that the former has the greatest prognostic significance. Of the various genes mapping to the commonly deleted sequence, the best candidate as a 'target' seems to be the p53 antioncogene. We applied our methods of detection of the p53 protein in a series of 78 colorectal cancers stored in a tumour bank from 1985 to 1989, for which the median follow-up was 42 months. Nuclear-attached p53 was quantified by flow cytometry and soluble p53 was assayed by ELISA. Both assays used a monoclonal antibody considered to be specific for a conformational epitope present only on the mutated protein. Fifty of the 78 tumours (64%) were found to present significant levels of p53 attached to the nucleus. A further two tumours contained high levels of p53 only in their soluble fraction. Thus, 52 out of 78 cancers (67%) were considered to be positive for p53. The p53 content correlated with 17p loss ($P < 0.002$), hyperdiploid DNA content ($P < 0.001$) and tumour site ($P < 0.03$), but not Dukes' stage ($P = 0.15$). p53 negative cases had a better overall survival than p53 positive ones ($P < 0.03$). When the 14 stage D tumours were excluded from the analysis, p53 was no longer significantly predictive of survival ($P < 0.07$), but remained predictive of recurrence ($P < 0.02$) and metastasis ($P < 0.03$). Multivariate analysis was not performed because of the small number of cases. Overall, disease-free and metastasis-free survival were compared to the positivity obtained either with pAb 421 and/or 1801 or pAb 240 since all three were used in the flow cytometric analysis, defining subsets of 421 –, 1801 + and 421 –, 1801 –, 240 +. The presence of nuclear protein presenting the mutation-specific epitope, recognised by pAb 240, was found to be the most discriminant. It must be noted that univariate survival analysis demonstrated that more than 80% of patients with p53-negative tumours were alive at 3 years *vs* less than 50% in the p53-positive group. A large prospective study should be conducted to define the exact prognostic significance of the p53 content of colorectal carcinomas.

An increasing body of evidence implicates the cellular anti-oncogene p53 in the development of human colorectal malignancy. In parallel to the discovery of the recurrent loss of the short arm of chromosome 17 (17p) by cytogenetic analysis (Muleris *et al.*, 1985; 1987), allelic losses were described, with probes specific for the short arm of chromosome 17, as well as for other chromosome segments (Fearon *et al.*, 1987; Law *et al.*, 1988; Delattre *et al.*, 1989; Vogelstein *et al.*, 1989; Fearon *et al.*, 1990). The deletions on 17p were found to encompass a common region containing the p53 gene (Baker *et al.*, 1989) and mutations have been described in a number of cancer specimens (Nigro *et al.*, 1989). More recently, it was shown that the transfection of a normal p53 gene in colon carcinoma cells inhibited their growth (Baker *et al.*, 1990).

Data concerning the expression of the protein have been obtained by immunochemical methods. Thus, a number of different groups have reported detection of p53 immunoreactivity in 42–54% of surgical specimens of colorectal cancer. pAb 421 was the most frequently used anti-p53 monoclonal antibody (Crawford *et al.*, 1984; Van den Berg *et al.*, 1989; Remvikos *et al.*, 1990), but there has been one recent report about immunodetection with pAb 1801 (Purdie *et al.*, 1991) and a small series using pAb 240 (Rodrigues *et al.*, 1990).

Mutations in the gene have been shown to provoke common conformational changes recognised by specific monoclonal antibodies, defining pAb 246-reactive 'pseudo-wild type' or 'overtly mutant' protein which reacts with pAb 240 (Gannon *et al.*, 1990). The latter has been applied to breast cancer cell lines (Bartek *et al.*, 1990) and human lung carcinomas (Iggo *et al.*, 1990), confirming its specificity for the altered p53 protein.

The aim of the present study was to investigate the influence of the p53 content of cancer cells on the outcome of the disease. Two monoclonal antibodies (MAbs) recognising epitopes present on the wild-type protein (pAb 421: C-terminal and 1801: N-terminal) and the mutant-specific pAb 240 were used. Immunoreactivity was assayed on nuclear suspensions obtained by dissociation of surgical specimens of colorectal cancer and on the soluble fractions using a two point ELISA.

### Materials and methods

The breast cancer cell line MDA-MB231 was cultured in a 1:1 mixture of DMEM and HAM F12 with 10% foetal calf serum (FCS) and 2% glutamine. A-431 cells (squamous carcinoma of the vulva) and HeLa cells (carcinoma of the cervix) were cultured in DMEM with 10% FCS. HL-60, myeloid leukaemia cells were cultured in RPMI 1640 with 20% FCS. In all cases exponentially growing cultures were harvested as previously described (Hammel *et al.*, 1991) and stored in liquid nitrogen.

A-431 and MDA-MB231 cells, which have been shown to contain homozygous p53 mutations (Bartek *et al.*, 1990; Chen *et al.*, 1990), were used as positive controls, HeLa cells, in which the p53 protein has been reported to be undetectable (Matlashewski *et al.*, 1986), and HL60 cells that do not contain p53 mRNA consecutive to large deletions in the gene (Wolf & Rotter, 1985), were used as negative controls.

Seventy-eight specimens of colorectal adenocarcinoma, operated between November 1985 and December 1989 and stored in our tumour bank were included in the study. All tissue fragments from the periphery of the tumours as well as macroscopically normal mucosa were obtained immediately after operation and stored at −80°C for short term conservation or in liquid nitrogen. These included 2 stage A, 29 stage

Correspondence: Y. Remvikos, Laboratoire de Radiopathologie, Institut Curie, 26, rue d'Ulm, 75231 Paris, France.
Current addresses: *Service de Gastroentérologie, Hôpital Beaujon, Clichy, France; †Service des Maladies du foie et de l'appareil digestif, Hôpital Kremlin-Bicêtre, France.
Received 4 December 1991; and in revised form 25 March 1992.

B, 23 stage C and 14 stage D cancers. Nineteen were situated in the right colon, 35 in the left or sigmoid colon and 24 in the rectum.

pAb 421 monoclonal antibody (Oncogene Science) and mouse IgG1 and IgG2a immunoglobulins (Southern Biotech.) were purchased as pure solutions. pAb 1801 and 240 anti-p53 monoclonal antibodies in the form of hybridoma supernatants and the rabbit polyclonal antiserum were generously provided by Dr D. Lane.

Small tissue fragments were dissociated as previously described (Remvikos *et al.*, 1990). Nuclear and soluble fractions were separated by a 20 s centrifugation at 10,000 *g*. The nuclear suspensions were stained for FCM analysis, while the supernatants were used in the ELISA. The tumour nuclei were filtered on nylon sheets (50 $\mu$m mesh), washed once with PBS and separated into equal fractions before incubation with the various antibodies.

pAb 421 and isotypic controls were used at 10 $\mu g\ ml^{-1}$ and the hybridoma supernatants were diluted to 1/4. All antibodies were diluted in PBS 0.1% BSA. The samples were incubated in 200 $\mu$l of the antibody dilution (1 h, room temperature), rinsed in PBS and resuspended in 200 $\mu$l of FITC-conjugated goat anti-mouse immunoglobulins (Southern Biotech.) diluted to 1/70 and incubated for a further 30 min. Free fluorescent antiserum was eliminated by centrifugation and the nuclei were counterstained with propidium iodide (25 $\mu g\ ml^{-1}$) and stored on ice until analysis.

Seventy-three tumours were analysed on a FACSTAR (Becton-Dickinson), equipped with a doublet discrimination module and five were part of the previously published series (Remvikos *et al.*, 1990). Settings were log for green fluorescence (p53) and linear for red fluorescence (DNA). Graphical representations and statistics were performed with the Consort 30 software. The fluorescence indices, taking into account the difference between total and non-specific fluorescences measured by FCM, were established for every case. The threshold value for positivity was 0.50, as previously described (Remvikos *et al.*, 1990).

Frozen sections from 16 tumours and paraffin sections from 46 tumours were available for immunohistochemistry. Endogenous peroxidase activity was inhibited by incubation with methanol + $H_2O_2$ (0.5%) for 15 min. Sections were incubated with anti-p53 polyclonal antibody CM1 at a dilution of 1:1000 for 1 h and the staining was revealed using the LSAB kit (DAKO) with diaminobenzidine as the substrate. Haematoxylin was used to counterstain nuclei.

A 2-point ELISA was used to detect p53 in the soluble fractions. The microtitre wells were incubated with anti-p53 polyclonal antibody CM1 at a dilution of 1:1000 for 2 h, and they were rinsed once with PBS-BSA (0.5%). The wells were saturated with PBS-BSA (0.5%) for 1 h, and were rinsed three times with PBS-BSA (0.1%) + Tween 20 (0.1%). The soluble fractions from tumours were distributed into the wells and incubated overnight at 4°C. After three rinses, the wells were incubated with pAb 240 at a dilution of 1:10 for 2 h at 37°C, and next with peroxidase-conjugated anti-mouse IgG1 at a dilution of 1:3000 for 1 h at 37°C. Background levels were established in the absence of sample or with samples

**Figure 1** Bivariate DNA/immunofluorescence plots of nuclei stained with isotypic control (**a**), pAb 1801 (**b**), pAb 421 (**c**), and pAb 240 (**d**).

incubated with mouse IgG1 (Southern) at 1 $\mu g\,ml^{-1}$ instead of pAb 240. O-phenylenediamine (ABBOTT) was used as the coloured substrate of peroxidase. The staining was stabilised with 4N sulphuric acid and quantified with a TEKAN LP500 at 492 nm.

Differences in survival were tested using the log-rank test and actuarial survival curves were plotted according to the Kaplan-Meier method.

### Results

An increase in immunofluorescence was observed for some nuclear suspensions from cancer specimens, incubated in the presence of anti-p53 monoclonal antibodies. For the diploid cancer shown in Figure 1, pAb 421 and 1801 produced similar shifts, while pAb 240 produced a somewhat weaker one. Six cases showed reproducible 1801 and 240 positivity, while the fluorescence histogram for 421 was indistinguishable from that of the isotypic control (Figure 2). A further seven cases were shown to react only with pAb 240. As for the cancer shown in Figure 3, the majority of the nuclei in the suspensions of six of the seven cases had hyperdiploid DNA content. The increase in fluorescence for pAb 240 was observed only for the hyperdiploid component. In some cases, the staining patterns presented some heterogeneity. For the case shown in Figure 4, pAb 421 (not shown) or pAb 1801 staining revealed both positive and negative cells, while the great majority of cells were positive with pAb 240. The difference was particularly striking for the cells with S-phase content. Finally, one cancer showed an increased fluorescence only for pAb 1801. In this last case, the fluorescence index for pAb 240 was 0.36, i.e. below the threshold value.

The three MAbs were applied to 73/78 surgical specimens. Five more cases from our first study (Remvikos et al., 1990) which were positive for pAb 421 were included in the survival analysis. Thus 44/73 (60%) of the colorectal adenocarcinomas were considered to the positive for pAb 240, compared to 38/73 (52%) for pAb 1801 and 36/78 (46%) for pAb 421. There were six cases with 421 –, 1801 +, 240 + phenotype, seven with 421 –, 1801 –, 240 + and one with 421 –, 1801 +, 240 –.

The soluble fractions were assayed separately by a 2-point ELISA, using the polyclonal antiserum as 'capture' antibody and pAb 240 as the 'revealing' antibody. The sensitivity of the assay was determined by using serial dilutions of A-431 and MDA-MB231 cells; p53 was measurable down to 200,000 cells/well. Thus, by using approximately $5.10^6$ cells/well for the soluble fractions it would be possible to measure down to 1/25th of the p53 expressed in the two cell lines. Background levels were established using HeLa and HL-60 cells for which the signals obtained did not exceed the control values (OD = 0.16 ± 0.05). If tumour fragments produced an insufficient number of cells (less than $3.10^6$ cells) or the control histograms showed less than 30% non-diploid cells, ELISA was considered non-informative.

High levels of p53 (OD > 0.35), compared to HeLa or HL-60 cells were obtained for 27/66 tumours (41%). The

**Figure 2** Flow cytometric results for an aneuploid tumour for the left colon, negative for pAb 421 (**b**), relative to isotypic control (**a**), weakly positive for pAb 1801 (**c**) and distinctly positive for pAb 240 (**d**).

**Figure 3** DNA histogram (upper panel) for a tumour of the rectum presenting a major peak of aneuploid tumour cells (right peak) and a minor peak (left peak) of presumably normal cells. Regions were set to select the diploid and aneuploid cells in order to analyse separately their immunofluorescence. Overlay of the immunofluorescence distributions for the diploid (middle panel) and aneuploid cells (lower panel), incubated with isotypic control (——), pAb 1801 (– –) or pAb 240 (·····). A significant shift was observed for the aneuploid cells and only after incubation with pAb 240.

**Figure 4** p53/DNA FCM results for a tumour of the rectum (DNA index 1.15). Equivalent results were obtained with pAb 421 (not shown) and pAb 1801 (**b**), relative to isotypic control (**a**). It must be noted that a more homogenous staining pattern was obtained with pAb 240 (**c**), compared to the two other anti p53 monoclonal antibodies. Extracts from this particular tumour were repeatedly negative in the ELISA.

remaining 12 cancers included the five pAb 421+ cases from the previous study and seven cases for which the assay was considered to be non informative, because of insufficient yield of tumour nuclei from the fragments. Although FCM and ELISA results showed a good correlation, a number of different situations were observed. A high content of nuclear-attached p53 was demonstrated by FCM for the case shown in Figure 4, while no p53 was found in the soluble fraction. On the other hand, the case presented in Figure 5 for which FCM analysis showed similar levels of nuclear-attached p53 to the previous case, produced one of the strongest signals in the ELISA.

Only two of the 27 ELISA-positive cases were entirely negative on FCM. One of them presented positive nuclei on immunohistochemical analysis (Figure 6) and the other one only showed faint cytoplasmic staining (not shown).

The p53 content of the 78 cancers was compared to the other biological parameters (17p loss and DNA content), as well as pathological stage and tumour site (Table 1). No significant correlation was found with Dukes' stage ($P=0.15$), but a highly significant correlation was obtained with DNA index $>1.30$ ($P<0.0001$) as well as 17p loss ($P<0.002$). p53 content was also significantly different when it was compared to tumour site ($P<0.03$), with a considerably reduced incidence in the proximal colon (42.1% positive cases) compared to an average of 74.8% in the distal segments.

The median follow-up for the 78 patients was 42 months. Actuarial overall survival curves according to a low or high p53 content are shown in Figure 7 (top panel). A high p53 content was found to be significantly linked to cancer mortality ($P<0.03$). The analysis was repeated after exclusion of the 14 stage D patients (not shown); in this case the difference did not reach statistical significance ($P<0.07$). The p53 content (for the non-metastatic patients) was found to be significantly predictive of disease-free (Figure 7: middle panel, $P<0.02$) and metastasis-free survival (Figure 7: bottom panel, $P<0.03$).

Since the p53 content was established by two methods, we were able to test its prognostic influence in different subgroups, according to MAb positivity or FCM *vs* ELISA. As

**Figure 5** Similar representations to those shown in Figure 4 for a diploid tumour of the sigmoid, FCM-positive with all three monoclonal antibodies and high positive in the ELISA.

**Figure 6** Section of a FCM-negative, ELISA-positive tumour, presenting distinctly stained nuclei.

**Table I** p53 expression in different subgroups

| | *p53−* | *p53+* | |
|---|---|---|---|
| Dukes ploidy 17p site | | | |
| A&B | 17 (40.5%) | 25 (59.5%) | $P = 0.15$ |
| C&D | 9 (25.0%) | 27 (75.0%) | |
| <1.30 | 19 (59.4%) | 13 (40.6%) | $P < 0.0001$ |
| >1.30 | 7 (15.2%) | 39 (84.8%) | |
| Normal | 11 (68.7%) | 5 (31.3%) | $P < 0.002$ |
| Lost | 5 (20.0%) | 20 (80.0%) | |
| RC | 11 (57.9%) | 8 (42.1%) | |
| LC | 10 (28.6%) | 25 (71.4%) | $P < 0.03$ |
| REC | 5 (20.8%) | 19 (79.2%) | |

shown in Table II, the most discriminant factor was found to be FCM-defined pAb 240 positivity (nuclear attached p53).

### Discussion

In our study, FCM analysis was used as a very sensitive method to investigate the p53 attached to the nuclei of colorectal carcinomas. Also, the use of monoclonal antibodies specific to the C-terminal (pAb 421) or N-terminal (pAb 1801) part of the molecule allowed us to demonstrate the existence of a minor subgroup for which the pAb 421 epitope was absent. More surprisingly, in seven cases there was evidence of reactivity exclusively with pAb 240. Since six of these cases had a hyperdiploid DNA content, the separate analysis of immunofluorescence for the diploid and hyperdiploid components, revealed that the increase in fluorescence was observed only for the hyperdiploid (presumably malignant) cells. Therefore, it seems improbable that this finding could be artefactual.

One of the possible criticisms concerning the FCM method was that the protein would have to remain attached to the nucleus throughout the processing. In a previous study we have shown that the use of whole cells released from fresh surgical specimens provided results similar to those obtained on nuclear suspensions from frozen fragments of the same cancers (Hammel *et al.*, 1991). However the effect of the fixation and permeabilisation procedure prior to application of the antibodies cannot be accounted for. We therefore developed a 2-point ELISA in order to quantify p53 in the soluble fractions of the initial dissociation step. The 'revealing' antibody in this case was also the mutated p53-specific pAb 240.

**Figure 7** Actuarial survival curves obtained according to the Kaplan-Meier method. The Mantel-Cox test was used for the differences between groups. Upper panel: overall survival (including stage D patients). Middle panel: disease-free survival and lower panel: metastasis-free survival according to p53 content, for patients with stage A to C2 tumours.

**Table II** p53 content and survival at 3 years

| *p53* | | | *% Disease free* | *% W/O metastasis* | *% Alive* |
|---|---|---|---|---|---|
| Nuclear | 421/1801 | − | 73 | 80 | 84 |
| | | + | 33 | 59 | 46 |
| | | | $P<0.02$ | $P<0.1$ | $P<0.005$ |
| | 240 | − | 83 | 92 | 88 |
| | | + | 35 | 54 | 45 |
| | | | $P<0.005$ | $P<0.02$ | $P<0.007$ |
| Nuclear and soluble | 240 | − | 82 | 92 | 86 |
| | | + | 38 | 57 | 48 |
| | | | $P<0.02$ | $P<0.03$ | $P<0.03$ |

One of the advantages of FCM is that by using additional parameters such as DNA content, the analysis can be selectively performed on cancer cells. ELISA, like all homogenate-based methods depends on the proportion of malignant cells in each fragment. For this reason, an aliquot of the initial suspension, because separation of the nuclear and soluble fractions, was stained with propidium iodide. Thus, the proportion of cancer cells can be easily determined, provided that the DNA index is >1.10 (70% of the present series). In a previous study comparing FCM and cytogenetic findings, we discussed this point in relation to the validity of FCM analysis in general (Remvikos *et al.*, 1988).

By comparing the FCM and ELISA results it was shown that three pAb 421+, eight pAb 1801+ and 11 pAb 240+ tumours based on the nuclear fractions, had no immunoreactivity p53 in their soluble fractions. Although sensitivity could have been one explanation, in favour of FCM, examples were presented with equivalent FCM positivity and highly divergent ELISA results. Thus it can be hypothesised that the balance of nuclear attached and soluble p53 in individual cases, may reflect particular biochemical properties possibly consecutive to gene mutations. Furthermore, no nuclear-attached p53 was demonstrated by FCM for two tumours which produced strong signals on ELISA. Immunohistochemical detection of the protein in paraffin embedded sections revealed positive nuclei for only one of them. The reasons why despite the nuclear localisation of the protein in the tissue section, no protein was detected in the nuclear suspensions prepared repeatedly from the frozen tumour fragments, is at present unclear. On the other hand, only weak cytoplasmic staining was observed for the remaining case. Cytoplasmic staining has been reported for breast (Bartek *et al.*, 1991), lung cancers (Iggo *et al.*, 1990) and melanomas (Stretch *et al.*, 1991).

The present study concluded that a high proportion of human colorectal cancers (67%) express increased levels of p53 immunoreactive with the mutated protein-specific monoclonal antibody pAb 240. However, the association with gene mutations has yet to be established. A number of colon or lung cancer cell lines have been described in which p53 protein can behave as mutant, while no evidence of mutations could be obtained (Rodrigues *et al.*, 1990; Lehman *et al.*, 1991). Thus, a better understanding of the properties of the p53 variants in colorectal cancers will be achieved after characterisation of the molecular events at the gene level.

Mutations of the p53 gene have been shown to be related to the loss of one allele of the short arm of chromosome 17 (Baker *et al.*, 1990). As expected, a similar correlation was found between 17p loss and increased levels of p53 protein displaying a mutation-specific epitope. We also observed a correlation between an increased p53 content and a DNA index >1.30. It must be stressed that a DNA index >1.30 has been previously found to be significantly linked with 17p loss (Delattre *et al.*, 1989). Thus it seems that the three parameters, 17p loss, high p53 protein content and hyperdiploid DNA content are closely interrelated.

In a series of 78 colorectal tumours, with a median follow-up of 42 months, we have shown that p53 was predictive of disease-free and overall survival. FCM-defined pAb 240 positivity was shown to be the most discriminant in terms of prognosis. This finding could be explained by the fact that recurrences occur in the high p53 group irrespective of the presence of the terminal epitopes. Recently, Scott *et al.* concluded on the absence of prognostic significance of nuclear p53 (Scott *et al.*, 1991). In their study, pAb 421 was used to stain p53 on frozen sections. These negative results could be due to the inability to obtain sufficient information concerning the presence of p53 with this particular MAb.

Because the results concerned only a short series of patients, we decided not to perform multivariate analysis. The small number of events in the low p53 group makes the statistical tests relatively unreliable. Nonetheless, the proportion of patients free of disease in the low p53 group was strikingly high (>80%), compared to that in the high p53 group (<50%).

Two different studies have concluded on the independent prognostic significance of 17p loss in colorectal cancers (Kern *et al.*, 1989; Laurent-Puig *et al.*, 1991). If we consider the information concerning DNA content, another prognostic factor according to some reports (Kokal *et al.* 1986; Jones *et al.*, 1988), then with the addition of a high p53 protein content, there are three potentially valuable biological prognostic parameters for colorectal cancers. However, as all three are closely correlated, only a large prospective study will be able to define their respective values for clinical purposes.

Despite the fact that genetic alterations of the p53 gene are probably among the most frequently occurring in human cancers (reviewed in Holstein *et al.*, 1991), this is the first time that the high content of a protein in the mutant conformation (defined immunologically) was found to influence patient outcome. The characterisation of the mutations might allow us to determine whether these are simply associated with the loss of its antioncogenic function or if the increased expression of the 'mutated' protein is indeed the key event. In the latter case, it can be hypothesised, at least for colorectal cancers, that p53 mutations can lead a step further from the recessive antioncogene scheme (Levine *et al.*, 1991), since some mutations might confer 'oncogenic' properties to the protein.

### References

BAKER, S.J., FEARON, E.R., NIGRO, J.M., HAMILTON, S.T., PRISSINGER, A.C., JESSUP,J.M., VAN THUINEN, P., LEDBETTER, D.H., BARKER, D.F., NAKAMURA, Y., WHITE, R. & VOGELSTEIN, B. (1989). Chromosome 17 deletions and p53 gene mutations in colorectal carcinomas. *Science*, **244**, 217–221.

BAKER, S.J., MARKOWITZ, S., FEARON, E.R, WILSON, J.K. & VOGELSTEIN, B. (1990). Suppressor of human colorectal carcinoma cell growth by wild p53. *Science*, **249**, 912–915.

BAKER, S.J., FREISINGER, A.C., MILBURN JESSUP, J., PARASKEVA, C., MARKOWITZ, S., WILSON, J.K.V., HAMILTON, S. & VOGELSTEIN, B. (1990). p53 gene mutations occur in combination with 17p allelic deletion as late events in colorectal tumourigenesis. *Cancer Res.*, **50**, 7717–7722.

BARTEK, J., IGGO, R., GANNON, J. & LANE, D.P. (1990). Genetic and immunochemical analysis of mutant p53 in human breast cancer cell lines. *Oncogene*, **5**, 893–899.

CHEN, P.-L., CHEN, Y., BOOKSTEIN, R. & LEE, W.-H. (1990). Genetic mechanisms of tumor suppression by the human p53 gene. *Science*, **250**, 1576–1579.

CRAWFORD, L.V., PIM, D.C. & LAMB, P. (1984). The cellular protein p53 in human tumors. *Mol. Cell. Med.*, **2**, 261–272.

DELATTRE, O., OLSCHWANG, S., LAW, D.J., MELOT, T., REMVIKOS, Y., SALMON, R.J., SASTRE, X., VALIDIRE, P., FEINBERG, A.P. & THOMAS, G. (1989). Multiple genetic alterations in distal and proximal colorectal cancer. *Lancet*, **ii**, 353–356.

FEARON, E.R., HAMILTON, S.R. & VOGELSTEIN, B. (1987). Clonal analysis of human colorectal tumors. *Science*, **238**, 193–197.

FEARON, E.R. & VOGELSTEIN, B. (1990). A genetic model for colorectal tumorigenesis. *Cell*, **61**, 759–767.

GANNON, J.V., GREAVES, R., IGGO, R. & LANE, D.P. (1990). Activating mutations in p53 produce a common conformation effect. *EMBO J.*, **9**, 1595–1602.

HAMMEL, P.R., BEUVON, F.X., SALMON, R.J. & REMVIKOS, Y. (1991). Immunochemical evidence of a mutated p53 protein expressed in human colorectal adenocarcinoma. *Gastroenterol. Clin. Biol.*, **15**, 529–535.

HOLSTEIN, M., SIDRANSKY, D., VOGELSTEIN, B. & HARRIS, C.C. (1991). p53 mutations in human cancers. *Science*, **253**, 49–53.

IGGO, R., GATTER, K., BARTEK, J. & LANE, D. (1990). Increased expression of mutant forms of p53 oncogene in primary lung cancer. *Lancet*, **335**, 675–679.

KERN, S.E., FEARON, E.R., TERSMETTE, K.W.F., ENTERLINE, J.P., LEPPERT, M., NAKAMURA, Y., WHITE, R., VOGELSTEIN, B. & HAMILTON, S.R. (1989). Clinical and pathologic associations with allelic loss in colorectal carcinomas. *JAMA*, **261**, 3099–3103.

KOKAL, W., SHEIBANI, K., TERZ, J. & HARADA, R. (1986). Tumor DNA content in the prognosis of colorectal carcinoma. *J. Am. Med. Assoc.*, **255**, 3123–3217.

JONES, D.J., MOORE, M. & SCOFIELD, P.F. (1988). Refining the prognostic significance of DNA ploidy in colorectal cancer; a prospective flow cytometry study. *Int. J. Cancer*, **41**, 206–210.

LAURENT-PUIG, P., OLSCHWANG, S., DELATTRE, O., REMVIKOS, Y., ASSELAIN, B., MELOT, T., VALIDIRE, P., MULERIS, M., GIRODET, J., SALMON, R.J. & THOMAS, G. (1992). Survival and somatically acquired alterations in colorectal cancer. *Gastroenterology*, (in press).

LAW, D., OLSCHWANG, S., MONPEZAT, J.P., LEFRANCOIS, D., JAGELMAN, D., PETRELLI, N.J., THOMAS, G. & FEINBERG, A. (1988). Concerted nonsynthenic allelic loss in human colorectal carcinoma. *Science*, **241**, 961–963.

LEHMAN, T.A., BENNET, W.P., METCALF, R.A., WELSH, J.A., MODALI, R.V., ULRICH, S., ROMANO, J.W., APELLA, E., TESTA, J.R., GERWIN, B.J. & HARRIS, C.C. (1991). p53 mutations, ras mutations and p53-heat shock 70 protein complexes in human lung carcinoma cell lines. *Cancer Res.*, **51**, 4090–4096.

LEVINE, A.J., MOMAND, J. & FINLAY, C.A. (1991). The p53 tumor suppressor gene. *Nature*, **351**, 453–456.

MATLASHEWSKI, G., BANKS, L., PIM, D. & CRAWFORD, L. (1986). Analysis of human p53 protein in normal and transformed cells. *Eur. J. Biochem.*, **154**, 665–672.

MULERIS, M., SALMON, R.J., ZAFRANI, B., GIRODET, J. & DUTRILLAUX, B. (1985). Consistent deficiencies of chromosome 18 and of the short arm of chromosome 17 in eleven cases of human large bowel cancer: a possible recessive determinism. *Ann. Genet.*, **28**, 206–213.

MULERIS, M., SALMON, R.J., DUTRILLAUX, A.M., VIELH, P., ZAFRANI, B., GIRODET, J. & DUTRILLAUX, B. (1987). Characteristic chromosomal imbalances in 18-near-diploid colorectal tumors. *Cancer Genet. Cytogenet.*, **29**, 289–301.

NIGRO, J.M., BAKER, S.J., PREISINGER, A.C., JESSUP, J.M., HOSTETTER, R., CLEARY, K., BIGNER, S.H., DAVIDSON, N., BAYLIN, S., DEVILEE, P., GLOVER, T., COLLINS, F.S., WESTON, A., MODALI, R., HARRIS, C.C. & VOGELSTEIN, B. (1989). Mutations in the p53 gene occur in diverse tumour type. *Nature*, **342**, 705–708.

PURDIE, C.A., O'GRADY, J., PIRIS, J., WYLLIE, A.H. & BIRD, C.C. (1991). p53 expression in colorectal tumors. *Am. J. Pathol.*, **138**, 807–813.

REMVIKOS, Y., MULERIS, M., VIEHL, P., SALMON, R.J. & DUTRILLAUX, B. (1988). DNA content and genetic evolution of human colorectal adenocarcinoma. A study by flow cytometry and cytogenetic analysis. *Int. J. Cancer*, **42**, 539–543.

REMVIKOS, Y., LAURENT-PUIG, P., SALMON, R.J., FRELAT, G., DUTRILLAUX, B. & THOMAS, G. (1990). Simultaneous monitoring of p53 protein and DNA content of colorectal adenocarcinomas by flow cytometry. *Int. J. Cancer*, **45**, 450–456.

RODRIGUES, N.R., ROWAN, A., SMITH, M.E.F., KERR, I.B., BODMER, W.F., GANNON, J.V. & LANE, D.P. (1990). p53 mutations in colorectal cancer. *Proc. Natl Acad. Sci. USA*, **87**, 7555–7559.

SCOTT, N.P., SAGAR, P., STEWART, J., BLAIR, G.E., DIXON, M.F. & QUIRKE, P. (1991). p53 in colorectal cancer: clinicopathological correlations and prognostic significance. *Br. J. Cancer*, **63**, 317–319.

STRECH, J.R., GATTER, K.C., RALFKIAER, E., LANE, D.P. & HARRIS, A.L. (1991). Expression of mutant p53 in melanoma. *Cancer Res.*, **51**, 5976–5979.

VAN DEN BERG, F.M., TIGGES, A.J., SCHIPPER, M.E.I., DEN HARTOG-JAGER, F.C.A., KROE, W.G.M. & WALBOMERS, J.M.M. (1989). Expression of the nuclear oncogene p53 in colon tumours. *J. Pathol.*, **157**, 193–199.

VOGELSTEIN, B., FEARON, E.R., KERN, S.E., HAMILTON, S.R., PREISINGER, A.C., NAKAMURA, Y. & WHITE, R. (1989). Allelotype of colorectal carcinomas. *Science*, **244**, 207–211.

WOLF, D. & ROTTER, V. (1985). Major deletions in the gene encoding the p53 tumor antigen cause lack of p53 expression in HL-60 cells. *Proc. Natl Acad. Sci. USA*, **82**, 790–794.